# The (Broad-Sense) Genetic Correlations Among Four Measures of Inattention and Hyperactivity in 12 Year Olds

**DOI:** 10.1007/s10519-020-10002-2

**Published:** 2020-06-11

**Authors:** Conor V. Dolan, Eveline L. de Zeeuw, Tetyana Zayats, C. E. M. van Beijsterveldt, Dorret I. Boomsma

**Affiliations:** 1 Department of Biological Psychology, Netherlands Twin Register, Vrije Universiteit, Van der Boechorststraat 7-9, 1081 BT Amsterdam, The Netherlands; 2 Amsterdam Public Health Research Institute, Amsterdam, The Netherlands; 3 Analytic and Translational Genetics Unit, Department of Medicine, Massachusetts General Hospital and Harvard Medical School, Boston, MA USA; 4 Stanley Center for Psychiatric Research, Broad Institute of MIT and Harvard, Cambridge, MA USA

**Keywords:** Inattention, Hyperactivity, ADHD, Genetic correlation, Multivariate genetic model, Twin design

## Abstract

We estimated the genetic covariance matrix among four inattention (INATT) and four hyperactivity (HYP) measures in the classical twin design. Data on INATT and HYP symptom counts were obtained in mono- and dizygotic twin pairs (N = 1593) with an average age of 12.2 years (sd = .51). We analyzed maternal ratings of INATT and HYP based on the Conners’ Parent Rating Scale (CPRS), the Strengths and Weaknesses of ADHD-symptoms and Normal-behavior (SWAN), and teacher ratings based on the Conners' Teacher rating scale (CTRS) and the ASEBA Teacher Rating Form (TRF). Broad-sense heritabilities, corrected for the main effects of sex and for random teacher rater effects, were large (ranging from .658 to .912). The results reveal pervasive and strong broad-sense genetic effects on INATT and HYP phenotypes with the phenotypic covariance among the phenotypes largely due to correlated genetic effects. Specifically between 79.9 and 99.9% of the phenotypic covariance among the HYP measures, and between 81.0 and 93.5% of the INATT measures are attributable to broad-sense genetic effects. Overall, the present results, pertaining to the broad-sense heritabilities and shared genetic effects, support the current genome-wide association meta-analytic approach to identifying pleiotropic genetic variants.

## Introduction

Attention deficit hyperactivity disorder (ADHD) is a relatively common (prevalence ~ 5.3–7.1%; Polanczyk et al. 2014; Willcutt 2007; Thomas et al. 2015; Boomsma et al. 2010; Mahone and Denckla 2017) childhood-onset neurodevelopmental disorder, which characterized by notable and age-inappropriate levels of inattention (INATT) and hyperactivity/impulsivity (HYP). Children with ADHD are at increased risk of anxiety disorders, learning disabilities, language disorders (Efron et al. 2016), and aggressive behavior (Bartels et al. 2018). Twin studies have established that the broad-sense genetic contributions to variance in Attention problems and ADHD liability are relatively large (h_b_^2^ =  ~ 0.75). There is little evidence for common (or shared) environmental effects, from twin or adoption studies (Rietveld et al. 2003; Burt 2009; Nikolas and Burt 2010; Chang et al. 2013; Kan et al. 2014; Larsson et al. 2014; Faraone and Larsson 2019), or from a recent study (de Zeeuw et al. 2020) that assessed genetic nurturing effects by analyzing untransmitted polygenic scores.

Research into the genetics of the ADHD liability in children, whose behavior usually is assessed by difference informants (self, teacher, parents), is complicated by systematic effects due to response styles, rating biases, method effects stemming from differences in psychometric instruments, and the situation-dependency of children's behavior (Merwood et al. 2013; Kan et al. 2014). Consequently, twin correlations can display considerable variation (see Tables 2 and 3 in Nikolas and Burt 2010). Parental and teacher ratings of ADHD symptoms in the same children are correlated modestly ~ 0.25 to ~ 0.50 (e.g., Narad et al. 2015; Martel et al. 2015; Merwood et al. 2013). Notably, heterogeneity poses a challenge in genome-wide association meta-analyses (GWAMA), which rely on meta-analysis of results from genome-wide association (GWA) studies based on different phenotypic measures (e.g. Demontis et al. 2019). Different measures of the same phenotype may display substantial measure-specific genetic variance and variable genetic covariance among the different measures. The latter is an important parameter in GWAMA, as the power to detect the effect of a given genetic variant depends on the degree to which its effect is shared among the different phenotypic measures used.

As ADHD liability combines the HYP and INATT liabilities, any source of individual differences may be traced approximately to these liabilities. Twin studies of inattention and hyperactivity liabilities have revealed relatively large genetic contributions (Nikolas and Burt 2010; Ebejer et al. 2015), which include additive and non-additive (dominance) effects, and the absence of shared environmental effects. The correlations between phenotypic measures of HYP and INATT in childhood depend potentially on the assessment instrument and the rater, with correlations within instruments and within raters generally being larger than the correlations between instruments and raters (Nadder et al. 2001; Achenbach et al. 1987; Fedko et al. 2017). In the Twins Early Development Study (TEDS), McLoughlin et al. (2007; in 8 year olds) and Greven et al. (2011; in 12 year olds) demonstrated common genetic influences on inattention and hyperactivity. At age 12, the genetic correlation was 0.55, indicating that the two dimensions are substantially influenced by the same genes, but that there also are unique genetic effects. At age 8 years, the correlation was approximately 0.60 (0.62 in boys; 0.57 in girls). Bidwell et al. (2017), in a study in (nominally unrelated) adults using bivariate GCTA (Lee et al. 2012), reported a substantial genetic correlation between inattention and hyperactivity-impulsivity (r = 0.861).

The aim of the present study is to establish, in the classical twin model, the broad-sense genetic covariance between 4 measures of INATT and 4 measures of HYP, in 12 year old children. We consider maternal ratings of INATT and HYP based on the Conners’ Parent Rating Scale (CPRS; Conners et al. 1998) and on the Strengths and Weaknesses of ADHD symptoms and Normal behavior (SWAN; Swanson et al. 2012), and teacher ratings for INATT and HYP based on the Conners' Teacher rating scale (CTRS) and the ASEBA Teacher Rating Form (TRF; Achenbach et al. 2001, 2017). In total, we had at our disposal four ratings by mother and four ratings by teachers. We modeled the covariance matrix of the eight phenotypic measures by a ADE Cholesky decomposition, allowing for additive genetic (A) and dominance (D) influences, and unshared environmental influences (E). Our main goals is to determine the broad sense genetic contribution to the phenotypic covariances among the different measures of INATT and HYP. In the model specification, we take into account that twins may be assessed by the same or by different teachers.

## Methods

### Participants

The data were obtained from the Netherlands Twin Register (NTR) at the Vrije Universiteit Amsterdam (van Beijsterveldt et al. 2013; Willemsen et al. 2013; Ligthart et al. 2019). The NTR contains twin and family data, collected from 1987 onwards, relating to health and behavior. In longitudinal NTR surveys, parents were asked to complete questionnaires just after their twins were born, and subsequently at ages 2, 3, 5, 7, 9/10, and 12 years. After obtaining parental consent, teachers are asked to rate the twins at ages 7, 9/10, and 12. We selected data obtained from twins born between 1986 and 1994. The present sample includes data from 1593 twin pairs (48% males, average age of 12.2 years), consisting of monozygotic (MZ) male pairs (N = 268), dizygotic (DZ) male pairs (N = 263), MZ female pairs (N = 352), DZ female pairs (N = 241), and DZ opposite sex pairs (N = 469).

### Phenotypic Measures

We analyzed four INATT and four HYP measures. They were obtained using the following four instruments:

1. Strengths and Weaknesses of ADHD Symptoms and Normal Behavior Rating Scales (SWAN; Swanson et al. 2012), which contains 18 items; 9 measuring inattention, and 9 measuring hyperactivity. The response format is a 7-point scale. The SWAN phenotypic scores were reverse coded, as, in contrast to the other measures, a higher SWAN score implies fewer endorsed symptoms, and so a more favorable test score.
2. Conners’ Parent Rating Scale—Revised: Short Form (CPRS, Conners et al. 1998). The response format is a 4-point scale (ranging from 0 = not true at all/never to 3 = very much true/very often). The CPRS contains 27 items, which are distributed over four subscales (three are included in two scales): Oppositional (6 items), ADHD Index (12 items), inattention (6 items) and hyperactivity (6 items). The INATT and HYP scales were included in the present analyses.
3. ASEBA-Teacher Report Form (TRF). The TRF contains 13 items in two subscales: inattention (5 items) and hyperactivity (8) (Achenbach et al 2017). The response format is a 3-point scale (0 = not consistent/not at all, 1 = somewhat consistent/sometimes, 2 = very consistent/often).
4. Conners’ Teacher Rating Scale—Revised: Short Form (CTRS, Conners et al. 1998). This instrument includes 28 items, which are distributed over four subscales (one item is included in two scales): oppositional (5 items), ADHD index (12 items), INATT (5 items) and HYPER (7 items). We included the last two in the present study. The response format is same 4-point scale as the CPRS.

The SWAN and the CPRS were completed by the mothers, the CTRS and the TRF were completed by the teachers. The CPRS, CTRS and TRF differ from the SWAN in the type of judgement that is required from the rater: the CPRS, CTRS and TRF item response requires an absolute statement on a 3- or 4-point scale, whereas item response format of the SWAN requires a statement relative to the "average child" on a 7 point scale (i.e., ranging from 1 = far below average to 7 = far above average). This results in a different distribution of the measures of INATT and HYP: SWAN measures tend to follow normal distribution, while those of the other instruments are left censored and positively skewed.

The phenotypic scores were obtained by computing the average item score. A phenotypic score was coded as missing if 20% or more of item responses were missing in a given participant. If less than 20% of items was missing for a subscale, the missing items were imputed by substituting the participant's average item subscale score.

### Data Analyses

Analyses were carried out in the R program version 3.4.1 (R Core Team 2017) and in Mplus (Muthén and Muthén 1998–2010). We used R for data management and descriptives, and Mplus for genetic covariance structure modeling of the twin data (Martin and Eaves 1977; Posthuma et al. 2003; Franić et al. 2012; Rijsdijk and Sham 2002). The twin design is used to obtain estimates of genetic and environmental variance components contributing to the (co)variance among one or more phenotype. The twin design exploits the fact that MZ twins share two alleles identically by descent (IBD) at all loci, leading to correlations between additive and non-additive genetic factors of one. Assuming random mating, the DZ twins on average share one allele IBD at autosomal loci, leading to an average correlation of 0.5 between additive genetic factors. DZ twin share dominance variance if they share two alleles IBD. As 25% of DZ twin pairs share two alleles IBD, these twins share the dominance variance. However, averaged over DZ twin pairs (of which, 25% share 2 alleles IBD, and 75% share 0 or 1 allele IBD), DZ twins shared 25% of the dominance variance, which corresponds to a correlation of 0.25 between dominance factors. Based on the difference in expected genetic resemblance of MZ and DZ pairs, we can decompose the phenotypic variance into additive genetic variance (A), dominance variance (D) *or* shared environmental variance (C), and unshared environmental variance (E) components. In practice, the phenotypic twin correlations (r_MZ_ and r_DZ_) determine the choice initial choice of model. If r_MZ_ > 2*r_DZ_ (which is the case with the present phenotypes), one proceeds with an ADE model.

We initially analyzed the eight phenotypes separately in univariate models, and then proceeded with the multivariate modeling of the eight phenotypes simultaneously. The genetic covariance structure model is as follows. Let M denote the number of phenotypes in the model, and let **Σ**_MZ_ and **Σ**_DZ_ represent the (2*M)  ×  (2*M) phenotypic covariance matrices in the MZ and DZ samples. We carried out both univariate and multivariate analyses. As the univariate model (M = 1) is a special case of the multivariate model (M = 8), we present the latter. The phenotypic 16 × 16 covariance matrices **Σ**_MZ_ and **Σ**_DZ_ were decomposed as follows:
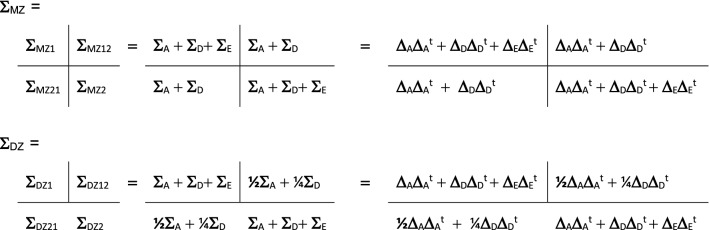


**Σ**_MZ_ and **Σ**_DZ_ have the indicated block structure, with the blocks representing covariance matrices within individual twins (e.g., **Σ**_MZ1_: the covariance between the 4 phenotypes) and between twin-1 and twin-2 (**Σ**_MZ21_ and **Σ**_DZ21_). **Σ**_A_, **Σ**_D_, and **Σ**_E_ are 8 × 8 covariance matrices, due to additive genetic, dominance, and unshared environmental effects, respectively. Below the matrices **Σ**_A_, **Σ**_D_, and **Σ**_E_ are parameterized by means of the Cholesky (or triangular) decomposition, i.e., **Σ**_A_ = **Δ**_A_**Δ**_A_^t^, where the 4 × 4 matrix **Δ**_A_ is lower triangular (similarly, **Σ**_D_ = **Δ**_D_**Δ**_D_^t^ and **Σ**_E_ = **Δ**_E_**Δ**_E_^t^). This parameterization has the advantage of ensuring that the A, D, and E covariance matrices are positive (semi-) definite. However, the results of interest are not the matrices **Δ**_A_, **Δ**_D_, and **Δ**_E_ (which we do not report), but rather the 8 × 8 matrices **S**_A_, **S**_D_, and **S**_E_, and their relative contributions to the 8 × 8 phenotype covariance of the INATT and HYP measures (**Σ**_A_ + **Σ**_D_ + **Σ**_E_). In the univariate analyses (M = 1), the matrices **Σ**_MZ_ and **Σ**_DZ_ are 2 × 2 covariance matrices, and **Σ**_A_, **Σ**_D_, and **Σ**_E_ are 1 × 1 matrices, containing simply the variances due to A, D, and E (and **Δ**_A_, **Δ**_D_, and **Δ**_E_ are standard deviations). We carried out the univariate analyses to obtain initial estimates of the univariate variance components (to inform the multivariate modeling), and to test sex differences in the magnitude of the A, D, and E variance components.

In addition to the estimation of the covariance matrices, we also fitted confirmatory common factor models to the 8 × 8 covariance matrices **Σ**_A_, **Σ**_D_, **Σ**_E_, i.e., the independent pathway model, a.k.a., the biometric factor model (see Martin and Eaves 1977; McArdle and Goldsmith 1990; Merwood et al. 2013; Kendler et al. 1987). In the case of the additive genetic model, the 8 × 8 covariance matrix (**Σ**_A_), was modeled by means of a 2 common factors model, in which the 4 HYP phenotypes and the 4 INATT phenotypes loaded on a common additive genetic HYP and a common additive genetic INATT model, respectively. We estimated the correlation between the common factors. The same applies to the dominance and unshared environmental covariance matrices, **Σ**_D_ and **Σ**_E_. The residuals of the phenotype (i.e., the part not explained by the common A, C, and E factors) were allowed to correlated between the twins. These correlations were free to vary over MZ and DZ twins, to accommodate possible genetic contributions to these residual terms.

In fitting the ADE Cholesky models, we included sex as a main effect in all models, and tested sex differences in variance components in the univariate models. In addition, we included an additional teacher rater factor (relevant to the teacher ratings using the TRF and CTRS), to accommodate the fact that twins from the same pair may be rated by the same teacher (Derks et al. 2006). Specifically, the teacher rater factor (common to the TRF and the CTRS scores) in twin 1 is correlated unity with teacher rater factor in twin 2, if the twins were in the same class, and so rated by the same teacher. If the twins were in differences classes, and so rated by different teachers, the correlation was fixed at zero.

### Data Transformation and Parameter Estimation

To fit the models to the data, we applied robust (normal theory) maximum likelihood (ML) estimation to Box-Cox transformed phenotypic data (except the SWAN data), and we report robust standard errors (i.e., the Mplus MLR estimator; Muthén and Muthén 1998–2010). We chose the Box-Cox transformation, because the parameter of this transformation can be optimized to render the data as normal as possible. Specifically, the Box-Cox transformation was carried out by pooling the data (by twin member and zygosity) and transforming the phenotypic data as follows: [(y + 1)^λ^ + 1]/λ, where y is the phenotype. The parameter λ was chosen to maximize the loglikelihood of the normal distribution. This transformation renders the data distributionally more suitable for normal theory ML estimation. The estimates of λ equaled − 1.474 (CPRS INATT), − 3.158 (CPRS HYP), − 1.053 (CTRS INATT), − 3.579 (CTRS HYP), − 2.737 (TRF INATT), and − 3.579 (TRF HYP). Following the transformation, which was applied to all phenotypes except the SWAN INATT and SWAN HYP, the data of all 8 phenotypes were linearly transformed so that the variances were approximately equal to one. This was done in the pooled data so that differences in phenotypic variances associated with sex, zygosity, and twin were retained. Rendering the variance to be about equal merely serves to facilitate raw data ML estimation. We considered the alternative approach of transforming the data to ordinal data (by creating, say, 3 point scales), and applying liability-threshold modeling (e.g., Derks et al. 2008) based on ML estimation. In the multivariate (16 × 16 phenotypic covariance matrices), this proved to be computationally intractable. An alternative is to use robust weighted least squares (WLS) estimation, applied to the polychoric correlation matrices (the MZ and DZ correlation matrices based on the ordinal data). However, WLS is based on pair-wise deletion, which is suboptimal in the present case, due to the extensive missingness (see below). We therefore resorted to robust ML estimation applied to the Box-Cox transformed data. To determine the dependency of the results on the Box-Cox transformation, we also consider the main results of interest obtained by analyzing the original, untransformed data. We provide these results in the appendices. Below we first present descriptive statistics, twin correlations and next the results of the univariate and multivariate genetic analyses.

## Results

The sample included 1593 twin pairs (48% male, 52% female) with an average age of 12.2 (standard deviation of 0.51). The year of birth ranged from 1986 to 1994, with 85% of the twins born in 1990 or 1991. The association of the phenotypes with age was negligible: the percentage of explained variance equaled 0.3% (CPRS HYP), 0.45% (CPRS INATT), 0.0 (CTRS HYP), 1.3% (CTRS INATT), 0.25% (TRS HYP), 0.5% (TRS INATT) and 6% (SWAN HYP) and 6% (SWAN INATT). We checked the effect of age on the ADE variance components of the SWAN phenotypes, but the effect of age was negligible. We therefore discarded age in the subsequent analyses. Of the 697 twin pairs that were rated by teachers, 57.2% were rated by the same teacher. Table 1 summarizes the missingness in the data. Because SWAN data were collected only in two NTR sub-projects (Polderman et al. 2007), the number of children with complete data (i.e., all eight phenotypes) is relatively small**.**

**Table 1:** Two-way and marginal representation of the number of missing values

Twin 2 members	Number of missing values in twin 1 members									Marginal twin 2
	0	1	2	3	4	5	6	7	8	
0	204	1	9	1	17	0	0	0	0	232
1	3	3	1	0	0	0	0	0	0	7
2	6	1	318	1	11	0	33	0	0	370
3	1	1	1	1	0	0	0	0	0	4
4	24	0	11	0	265	4	4	0	3	311
5	0	0	0	0	4	5	1	0	1	11
6	0	0	33	1	5	1	591	4	6	641
7	0	0	0	1	0	0	2	2	1	6
8	0	0	1	0	7	0	3	0	0	11
Marginal twin 1	238	6	374	5	309	10	635	6	11	

### Phenotypic Results

**Fig. 1:**
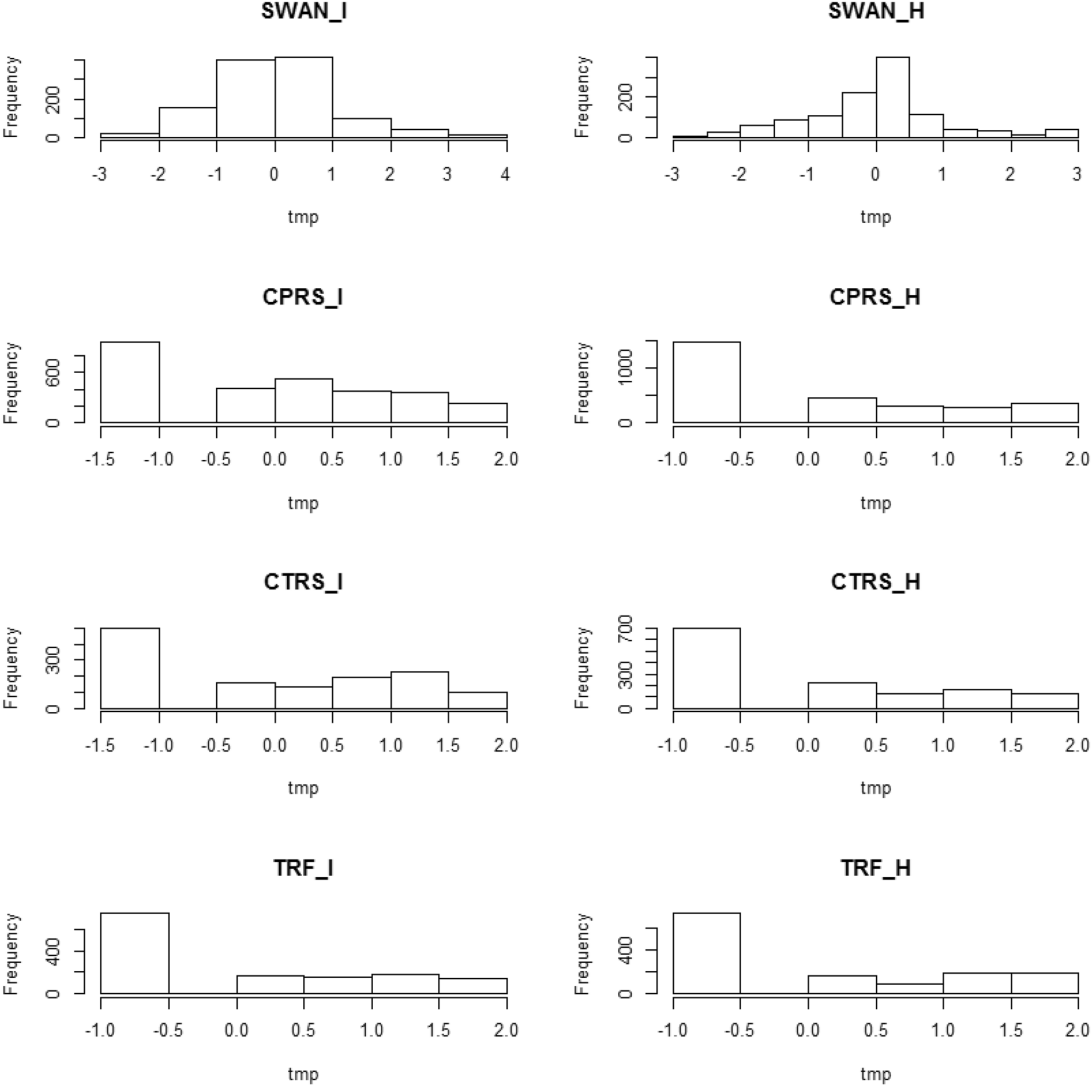
Histograms of the phenotypic data (pooled by sex and twin member). The CPRS, CTRS, and the TRF data were Box-Cox transformed. *I* inattention, *H* hyperactivity

Figure 1 shows histograms of the phenotype data. Table 2 contains descriptive statistics (mean, standard deviation, skewness and kurtosis). The ML estimates of the phenotypic 16 × 16 MZ and DZ correlation matrices are shown in “Appendix 1” Tables 7 and 8. The main effect of sex was consistently significant (p < 0.001) (see Table 3). The HYP twin correlations in Tables 7 and 8 are (r_MZ_ vs. r_DZ_): 0.91 vs. 0.46 (SWAN), 0.78 vs. 0.32 (CPRS), 0.64 vs. 0.25 (TRF), 0.60 vs. 0.31 (CTRS). The INATT twin correlations are: 0.85 vs. 0.40 (SWAN), 0.74 vs. 0.24 (CPRS), 0.63 vs. 0.16 (TRF), 0.72 vs. 0.41 (CTRS). The *within-instrument* phenotypic correlations between INATT and HYP are ~ 0.83 (SWAN), ~ 0.55 (CPRS), ~ 0.62 (TRF), and ~ 0.35 (CTRS). The *across-instrument* INATT correlations vary from ~ 0.31 to 0.56, and the *across-instrument* HYP correlations vary from ~ 0.77 to 0.20. Interrater (i.e., mother vs. teacher) correlations vary from ~ 0.29 to 0.54 (INATT) and ~ 0.20 to 0.38 (HYP). These values are consistent with the results of previous articles (e.g., Narad et al 2015; Martel et al. 2015).

**Table 2:** Summary statistics of transformed data by zygosity (MZ and DZ) and twin member (1 and 2)

Trait	N	Mean	St dev	Skewness	Kurtosis
MZ					
SWAN INATT 1	246	0.074	1.090	0.793	0.952
SWAN HYP 1	247	0.082	1.095	0.669	0.759
CPRS INATT 1	552	− 0.065	1.007	0.242	− 1.351
CPRS HYP 1	553	− 0.018	1.007	0.456	− 1.377
CTRS INATT 1	259	− 0.009	0.987	0.316	− 1.397
CTRS HYP 1	259	− 0.039	0.999	0.595	− 1.256
TRF INATT 1	277	− 0.041	0.986	0.585	− 1.375
TRF HYP 1	277	− 0.018	1.035	0.588	− 1.350
SWAN INATT 2	246	0.087	1.144	0.686	0.853
SWAN HYP 2	251	0.104	1.096	0.619	0.827
CPRS INATT 2	552	− 0.097	1.024	0.293	− 1.364
CPRS HYP 2	550	− 0.070	1.004	0.560	− 1.282
CTRS INATT 2	265	− 0.005	0.992	0.333	− 1.378
CTRS HYP 2	266	− 0.111	0.972	0.774	− 0.922
TRF INATT 2	282	− 0.090	0.969	0.666	− 1.272
TRF HYP 2	283	− 0.041	1.016	0.637	− 1.237
DZ					
SWAN INATT 1	246	− 0.091	0.911	0.155	0.343
SWAN HYP 1	247	− 0.088	0.885	− 0.165	0.812
CPRS INATT 1	552	0.015	1.014	0.146	− 1.349
CPRS HYP 1	553	− 0.035	1.015	0.506	− 1.329
CTRS INATT 1	259	− 0.041	0.975	0.333	− 1.347
CTRS HYP 1	259	− 0.043	0.980	0.598	− 1.190
TRF INATT 1	277	− 0.009	0.981	0.516	− 1.406
TRF HYP 1	277	− 0.052	1.015	0.624	− 1.282
SWAN INATT 2	246	− 0.022	0.901	0.216	1.039
SWAN HYP 2	251	− 0.065	0.954	− 0.048	0.676
CPRS INATT 2	552	0.006	1.032	0.204	− 1.362
CPRS HYP 2	550	− 0.026	1.026	0.514	− 1.328
CTRS INATT 2	265	0.084	0.983	0.210	− 1.357
CTRS HYP 2	266	− 0.011	0.983	0.530	− 1.273
TRF INATT 2	282	0.091	0.981	0.295	− 1.585
TRF HYP 2	283	0.029	1.034	0.494	− 1.434

Variation in sample size N is due to missingness

**Table 3:** Results of univariate modeling: estimates of fixed effect of sex, MZ and DZ correlations, and variance due to rater (i.e., same or different teacher), additive genetic (A) and dominance (D) effects, and unshared environment (E)

	β_sex_	R^2^	r_mz_	r_dz_	Rater	A	D	E	A + D
					r^2^	a^2^	d^2^	e^2^	a^2^ + d^2^
SWAN HYP	− 0.14 (0.032)	2.0%	0.899 (0.017)	0.364 (0.069)	–	0.852 (0.284)	0.063 (0.282)	0.085 (0.012)	0.915
CPRS HYP	− 0.23 (0.019)	5.3%	0.781 (0.024)	0.311 (0.032)	–	0.464 (0.134)	0.317 (0.131)	0.219 (0.014)	0.781
TRF HYP	− 0.29 (0.027)	8.6%	0.651 (0.045)	0.260 (0.051)	0.206 (0.053)	0.000^§^	0.548 (0.054)	0.246 (0.043)	0.548
CTRS HYP	− 0.29 (0.027)	8.8%	0.718 (0.053)	0.093 (0.094)	0.331 (0.047)	0.000^§^	0.484 (0.050)	0.185 (0.037)	0.484
SWAN INATT	− 0.17 (0.031)	2.8%	0.853 (0.022)	0.412 (0.064)	–	0.794 (0.258)	0.059 (0.253)	0.147 (0.017)	0.853
CPRS INATT	− 0.21 (0.019)	4.5%	0.735 (0.025)	0.239 (0.033)	–	0.221 (0.134)	0.514 (0.141)	0.265 (0.025)	0.735
TRF INATT	− 0.23 (0.028)	5.3%	0.670 (0.054)	0.05 (0.096)	0.242 (0.048)	0.000^§^	0.515 (0.050)	0.244 (0.048)	0.515
CTRS INATT	− 0.090 (0.028)	0.8%	0.733 (0.041)	0.370 (0.063)	0.193 (0.059)	0.591 (0.059)	0.000^§^	0.216 (0.034)	0.591

Robust standard errors are given in parentheses

Intercepts (not shown) and regression coefficients (β_sex_) were constrained to be equal over twins and zygosities. The parameters β_sex_ are standardized regression coefficients, i.e., interpretable as differences in standard deviation units (Cohen's d). R^2^ is the proportion of variance explained by the main effect of sex (coded 0 = boys; 1 = girls). The variance components (r^2^, a^2^, d^2^, and e^2^) are standardized (r^2^ + a^2^ + d^2^ + e^2^ = 1). a^2^ + d^2^ is the broad-sense heritability, h_b_^2^

^§^Estimated at zero (not fixed to zero)

### Univariate ADE Results

The twin correlations, main sex effects, and estimated standardized variance components, based on the univariate twin model, are shown in Table 3. The A variance component was estimated at zero in the case of TRF INATT and HYP, and CTRS HYP. Broad-sense heritabilities (standardized A + D variance components) ranged from 0.915 (SWAN HYP) to 0.484 (CTRS HYP). The absence of additive genetic variance is notable, as we expect dominance variance to be accompanied by additive genetic variance (Falconer and MacKay 1996). However, the large differences in twin correlations associated with childhood ADHD measures are well established, as is the presence of dominance in meta-analyses of twin correlations (Nikolas and Burt 2010; Jepsen and Michel 2006; Pingault et al. 2015). The teacher rater effects account for about 20% to 24% of the variance of TRF HYP, TRF INATT and CTRS INATT, and a relatively large 33% of the variance of CTRS HYP.

The regression coefficients (β_sex_ in Table 3) represent the main effect of sex on the phenotypes. Note that the regression coefficients are negative because sex is coded zero (males)/one (females), and males on average showed more ADHD symptoms. The variance explained by sex varies from ~ 1 to ~ 9%, which is associated with an effect size in standard deviation units (Cohen's d) of ~ 0.09 to ~ 0.29.

We tested sex differences in the magnitude of the A, D and E variance components, but found only two differences (given α = 0.01; see “Appendix 2” Table 9). These concerned the dominance variance of the SWAN HYP and the CPRS INATT. Given that the apparent sex differences were limited to two of the 24 variance components shown in “Appendix 2” Table 9, we did not consider sex moderation of variance components in the multivariate analyses. However, we did include in the multivariate analyses the main effect of sex.

### Multivariate ADE Results

Table 4 contains the estimated standardized A, D, and E variance components based on the multivariate Cholesky decomposition (corrected for the main effects of sex and for the teacher rater effect). Compared to those in Table 3, these results are more consistent with biometric theory, which predicts that the dominance components should be accompanied by additive genetic components (Falconer and McKay 1996). Still, the dominance components are relatively large in the case of TRF HYP (D: 0.454 vs A: 0.230), CTRS HYP (0.492 vs. 225), TRF INATT (0.482 vs 0.176). The broad-sense heritabilities are relatively large: from ~ 0.91 (SWAN HYP) to ~ 0.65 (TRF INATT). Ebejer et al. (2015) reported similar broad sense heritabilities for maternal SWAN ratings. The unshared environmental variance components are consequently relatively small (~ 0.09 to ~ 0.34). Note that the environmental variance components include a measurement error term, so that the effects of the true unshared environmental factors are actually smaller.

**Table 4:** Standardized variance components based on a multivariate Cholesky decomposition (corrected for teacher rater and sex)

	SWAN HYP	CPRS HYP	TRF HYP	CTRS HYP	SWAN INATT	CPRS INATT	TRF INATT	CTRS INATT
Additive genetic (A) Narrow sense h^2^	0.687 (0.118)	0.510 (0.115)	0.230 (0.101)	0.225 (0.144)	0.609 (0.082)	0.369 (0.099)	0.176 (0.068)	0.643 (0.092)
Non-additive genetic (D: dominance)	0.226 (0.225)	0.275 (0.120)	0.454 (0.150)	0.492 (0.185)	0.234 (0.076)	0.369 (0.107)	0.482 (0.115)	0.084 (0.060)
Broad-sense (A + D) h^2^	0.912 (0.014)	0.784 (0.023)	0.683 (0.081)	0.717 (0.090)	0.844 (0.021)	0.738 (0.047)	0.658 (0.070)	0.727 (0.063)
Unshared environmental (E)	0.088 (0.014)	0.216 (0.023)	0.317 (0.081)	0.283 (0.090)	0.156 (0.021)	0.262 (0.047)	0.342 (0.070)	0.273 (0.063)

Robust standard errors are given in parentheses

Table 5 contains the A, D, A + D, and E correlation matrices. The upper triangle of the A + D matrix in Table 5 contains the proportion of phenotypic (co-)variance explained by A and D. The diagonals contain the broad-sense heritabilities (information also given in Table 4), the off-diagonals in the upper triangle contain the proportion of phenotype covariance attributable to A + D. Similarly, the upper part of the E matrix in Table 5 contains the proportions of phenotypic (co-)variance explained by E. In terms of the model parameters collected in the matrices **Σ**_A_, **Σ**_D_, and **Σ**_E_, the upper triangle of the A + D and E matrices were calculated as (**Σ**_A_ + **Σ**_D_)/(**Σ**_A_ + **Σ**_D_ + **Σ**_E_) and **Σ**_E_/(**Σ**_A_ + **Σ**_D_ + **Σ**_E_), respectively.

**Table 5:** Genetic (A, D, A + D), and unshared environmental (E) correlation matrices based on the multivariate Cholesky decomposition

**A**	SWAN H	CPRS H	TRF H	CTRS H	SWAN I	CPRS I	TRF I	CTRS I
SWAN H	1.000							
CPRS H	0.357	1.000						
TRF H	0.369	0.159	1.000					
CTRS H	0.304	0.031	0.942	1.000				
SWAN I	0.995	0.401	0.314	0.231	1.000			
CPRS I	0.392	0.967	0.296	0.231	0.418	1.000		
TRF I	0.449	0.355	0.372	0.354	0.481	0.454	1.000	
CTRS I	0.373	0.288	0.552	0.590	0.377	0.449	0.952	1.000

**D**	SWAN H	CPRS H	TRF H	CTRS H	SWAN I	CPRS I	TRF I	CTRS I
SWAN H	1.000							
CPRS H	0.859	1.000						
TRF H	0.651	0.595	1.000					
CTRS H	0.643	0.704	0.824	1.000				
SWAN I	0.504	0.576	0.543	0.783	1.000			
CPRS I	0.436	0.214	0.509	0.542	0.810	1.000		
TRF I	0.489	0.357	0.925	0.754	0.640	0.741	1.000	
CTRS I	0.450	0.231	0.334	0.373	0.767	0.957	0.563	1.000

**A + D**	SWAN H	CPRS H	TRF H	CTRS H	SWAN I	CPRS I	TRF I	CTRS I
SWAN H	***0.912***	***0.888***	***0.999***	***0.926***	***0.922***	***0.847***	***0.930***	***0.936***
CPRS H	0.502	***0.784***	***0.799***	***0.794***	***0.907***	***0.896***	***0.882***	***0.909***
TRF H	0.450	0.361	***0.683***	***0.814***	***0.842***	***0.877***	***0.859***	***0.845***
CTRS H	0.413	0.359	0.862	***0.717***	***0.935***	***0.884***	***0.894***	***0.835***
SWAN I	0.866	0.454	**0.388**	0.452	***0.844***	***0.821***	***0.885***	***0.865***
CPRS I	0.394	0.641	0.414	0.409	0.553	***0.738***	***0.863***	***0.810***
TRF I	0.409	0.329	0.757	0.637	0.500	0.615	***0.658***	***0.772***
CTRS I	0.380	0.265	0.393	0.416	0.439	0.528	0.627	***0.727***

**E**	SWAN H	CPRS H	TRF H	CTRS H	SWAN I	CPRS I	TRF I	CTRS I
SWAN H	***0.088***	***0.112***	***0.001***	***0.074***	***0.078***	***0.153***	***0.070***	***0.064***
CPRS H	0.387	***0.216***	***0.201***	***0.206***	***0.093***	***0.104***	***0.118***	***0.091***
TRF H	0.002	0.254	***0.317***	***0.186***	***0.158***	***0.123***	***0.141***	***0.155***
CTRS H	0.169	0.283	0.460	***0.283***	***0.065***	***0.116***	***0.106***	***0.165***
SWAN I	0.549	0.207	0.248	0.116	***0.156***	***0.179***	***0.115***	***0.135***
CPRS I	0.384	0.239	0.143	0.144	0.471	***0.262***	***0.137***	***0.190***
TRF I	0.138	0.116	0.254	0.167	0.209	0.227	***0.342***	***0.228***
CTRS I	0.136	0.082	0.173	0.213	0.259	0.340	0.418	***0.273***

The upper triangular part of the matrices A + D and E contain the proportion of phenotypic variance (diagonals; broad-sense heritabilities in the case of the A + D matrix; bold italics underscored), and the proportion of phenotypic covariances (above the diagonal; bold italics) explained by A + D and E

**Fig. 2:**
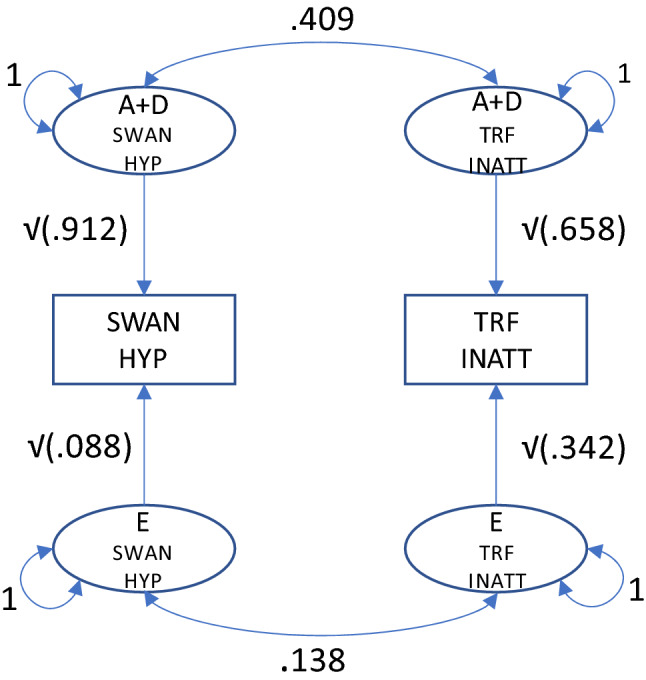
Bivariate completely standardized model (SWAN HYP and TRF INATT). The phenotypic correlation is r_ph_ = √.912*.409*√.658 + √.088*.138*√.342 =  ~ .340. The broad sense genetic contribution, expressed as a proportion, is (√.912*.409*√.658)/.340 = .316 /.340 =  ~ .930. The values .912, .658, .088 and .342 are given in Table 4. The values .409, .138, and .930 are given in Table 5. The expected phenotypic correlation (.340) is given in Table 6

The broad-sense genetic correlations among the HYP measures range from 0.359 to 0.862, with an average correlation of ~ 0.50. The broad-sense genetic correlations among the INATT measures range from 0.439 to 0.627, with an average correlation of ~ 0.55. The environmental correlations are consistently lower. The proportions of phenotypic covariance attributable to A + D are highly illuminating. We find that between 79.9 and 99.9% of the phenotypic covariance among the HYP measures, and between 81.0 and 93.5% of the INATT measures are attributable to A + D. Figure 2 explains the calculations involved in arriving at these percentages in the case of the SWAN HYP and the TRF INATT.

The broad-sense genetic correlations between INATT and HYP are 0.866 (based on the SWAN), 0.641 (CPRS), 0.757 (TRF) and 0.416 (CTRS). The corresponding unshared environmental correlations are again generally lower. The broad sense genetic factors (i.e., A + D) accounted for most of the phenotypic correlations. The proportions of the phenotypic covariance attributable to genetic factors (A + D) equal 92.2% (SWAN), 89.6% (CPRS), 85.9% (TRF), and 83% (CTRS). Based on these results (Tables 4 and 5), we see that pleiotropic genetic effects are the dominant source of phenotypic covariance among the different INATT and HYP measures (assessed with different instruments/raters), and between the INATT and HYP measures (assessed with the same instrument/rater). Table 6 contains the phenotypic correlations based on the results in Tables 4 and 5. These are easier to interpret than the four (MZ twin 1, 2; DZ twin 1, 2) sets of observed phenotypic correlations provided in the “Appendix 1” Tables 7 and 8.

**Table 6:** Model implied phenotypic correlations based on the Cholesky ADE model (calculated using the results in Tables 4 and 5)

Pheno	SWAN H	CPRS H	TRF H	CTRS H	SWAN I	CPRS I	TRF I	CTRS I
SWAN H	1.000							
CPRS H	0.478	1.000						
TRF H	0.355	0.331	1.000					
CTRS H	0.361	0.339	0.741	1.000				
SWAN I	0.824	0.407	0.350	0.376	1.000			
CPRS I	0.382	0.544	0.335	0.337	0.532	1.000		
TRF I	0.341	0.268	0.591	0.489	0.421	0.497	1.000	
CTRS I	0.330	0.220	0.328	0.360	0.397	0.478	0.561	1.000

### Results Based on the Untransformed Scales

The present results were obtained with the Box-Cox transformed data. It is well known that results obtained following non-linear transformation of the data can differ from those obtained with the untransformed data. This holds for the analyses of interaction (Eaves 2005), but also holds for main effects (Eaves et al. 1977). To evaluate the effects of this transformation on the results, we also analyzed the untransformed data. Tables 10 and 11 contain the same results as Tables 4 and 5, but based on the untransformed data. The decomposition of phenotypic variance into A and D components differed in details, with notable differences relating to the CPRS INATT and HYP. Specifically, in the results based on the original scales, we see a large D variance component (HYP D:0.721, A:0.094; INATT D:0.632, A:0.132), whereas in the results based on the transformed data, the A components are larger (HYP D:0.275, A:0.510; INATT D:0.369, A:0.369). However, we found that overall the estimated broad-sense genetic and unshared environmental correlations and variance components are quite similar when analyzing the transformed (Tables 10, 11) and untransformed data (Tables 4, 5). That is, while scaling issue are important, they have little bearing on the present conclusion.

Finally we attempted to fit the (independent) common factor model as described in the methods section. However, this model failed to converge. We attribute this to the extensive missingness (see Table 1) and to the variation in the additive genetic and unshared environmental correlations. Notably the additive genetic correlation between TRF HYP and SWAN HYP (0.002; see Table 5) is inconsistent with a common additive genetic factor. Similarly the unshared environmental correlation between the CTRS HYP and CPRS HYP is low (0.031; see Table 5), which is not compatible with a common unshared environmental factor. As an alternative, we fitted the two common factor model to the A + D correlation matrix (Table 5). In this approach, we obtained only the parameter estimates, but no standard errors or measure of model fit, and the results pertain only to the A + D correlation matrix. The standardized factor loadings on the common A + D factor HYP were 0.366 (SWAN HYP), 0.427 (CPRS HYP), 0.918 (TRF HYP), and 0.925 (CTRS HYP). The standardized factor loadings on the common A + D factor INATT factor were 0.476 (SWAN INATT), 0.680 (CPRS INATT), 0.901 (TRF INATT), and 0.704 (CTRS INATT). The correlation between the common A + D HYP and INATT factors equaled 0.75.

## Discussion

The aim of the present paper was to establish, using the classical twin design, the genetic covariance matrix of four measures of inattention (INATT) and four measures of hyperactivity (HYP) in ~ 12 year old children. The broad-sense heritabilities (A + D) of the HYP phenotypes (~ 68–91%) and INATT phenotypes are relatively large (65–84%). The broad-sense genetic correlations between INATT and HYP vary considerably: 0.86 (SWAN), 0.64 (CPRS), 0.75 (TRF) and 0.41 (CTRS). The correlation between the INATT and HYP common factors equaled 0.75 (as estimated in the confirmatory factor analysis of the A + D correlation matrix). However, the contributions of broad sense genetic factor to the phenotypic correlations are large. The phenotypic correlations are 0.82 (SWAN), 0.54 (CPRS), 0.59 (TRF), and 0.36 (CTRS). The broad sense genetic contributions to these phenotypic correlations are 92%, 89%, 85%, and 83%, respectively. Phenotypic correlations among the four HYP measures vary between 0.331 and 0.741. Broad sense genetic factors account for 68% to 99% of these phenotypic correlations. The large genetic contributions to the phenotypic correlations among the HYP and INATT measures support the view that ADHD has a strong genetic liability (Faraone and Larsson 2019).

In this study, the average A + D genetic correlation is 0.49 (min: 0.265, max: 0.866). The relevant correlations are given in the third panel of Table 5 (indicated by A + D). Since it does not equal one, the phenotypes of HYP and INATT are characterized by broad sense genetic components that are specific to them (e.g., Nadder et al. 2001; Kuntsi et al. 2014; Greven et al. 2011; Merwood et al. 2013; Faraone and Larsson 2019). We can see this in more detail in the results of the confirmatory factor analysis of the A + D correlation matrix. In this model, the common A + D HYP factor accounted for 14%, 18%, 85% and 85% of the broad sense genetic variance of SWAN HYP, CPRS HYP, TRF HYP, and CTRS HYP, respectively. The common A + D INATT factor accounted for 26%, 48%, 77% and 50% of the broad sense genetic variance of SWAN INATT, CPRS INATT, TRF INATT, and CTRS INATT, respectively. Thus, the phenotype specific genetic effects vary considerably, with the SWAN HYP and INATT having the largest genetic residual (86% and 74%), and TRF HYP, and CTRS HYP having the smallest (both 15%).

Overall, the present results, pertaining to the broad-sense heritabilities and shared genetic effects, support the current GWAMA approach to identifying pleiotropic genetic variants contributing to ADHD variance. Specifically, we found that the HYP and INATT measures show strong genetic correlations, irrespective of the instrument used to obtain these. This suggests that the genetic effects shared between INATT and HYP are likely to be captured in the GWAMA of ADHD, notwithstanding the expected (in a meta-analyses) variation in the psychometric instrument and method used to measure ADHD. It is possible that the moderating effects of rater, psychometric instrument, and situational factors introduce variation in genetic effect sizes. This may ultimately call for a random effects meta-analytic approach. We note, however, that Demontis et al. (2019) found no such random effects in their GWA meta-analysis of ADHD.

We note the following limitations of the present study. We did not consider contrast effects, although these have been demonstrated (e.g., Ebejer et al. 2015; Merwood et al. 2013; Nadder et al. 2001). In the univariate ADE twin model, the contrast effect is accommodated by including a reciprocal pathway between the twins phenotypes. Prior univariate twin modeling demonstrated that BIC favored the ADE model without contrast effects. BIC favored the ADE model with contrast effect in the case of SWAN INATT and TRF INATT. However the differences in BIC were small (2872 vs. 2871 and 2376 vs. 2375, respectively). In addition, an indication of contrast effects is larger DZ phenotypic variance than MZ phenotypic variance, which we do not see in these data (see Table 1). Finally, the absence of contrast effect in the NTR ADHD twin data was also demonstrated by Rietveld et al. (2003).

While the results are consistent with the literature, we acknowledge that estimates may be biased. Notably, the present results are based on the classical twin design, which is characterized by many strong assumptions (Eaves et al. 1977). These include the equal environment assumption, random mating, and absence of interplay, such as G-E correlation and GxE interaction. Any violation of these assumptions will introduce bias in the variance components. However, the effects of violations of these assumptions on the variance components, as estimated in the twin model, are well known (e.g., Purcell 2002). Specifically, AxE and DxE interaction contribute to E, and cor(AC) and cor(DC) contribute to C. As in the present results E is relatively small and C is all but absent, it is likely that the effects of these possible violations are negligible. AxC and r(AE) contribute to A, and DxC and r(DE) contribute to D. These may be relatively important given the large broad-sense heritabilities. In addition, a possible role of AxC and DxC interaction does not seem farfetched (e.g., see Hicks et al. 2009). Random mating is an important assumption, as it has a bearing on the additive genetic correlations in the DZ twin (0.5, given random mating). However, the spousal phenotypic correlation in Dutch ADHD data is low (~ 0.11; Boomsma et al. 2010), which suggests that assortative mating is not likely to be an appreciable source of bias. Ultimately, supplementing the twin model with linear combinations of measured genotypes (polygenic scores) associated with ADHD, as established in GWAMA, will provide the means to address issues relating to this interplay in the twin model.

## Appendix 1

See Tables 7 and 8.

**Table 7:** ML estimates of the MZ phenotypic correlation matrix corrected for main effects of sex

**MZ twin 1: within person**								
	SWANH1	CPRSH1	TRFH1	CTRSH1	SWANI1	CPRSI1	TRFI1	CTRSI1
SWANH1	1.00	0.30	0.27	0.29	0.83	0.30	0.23	0.37
CPRSH1	0.30	1.00	0.32	0.27	0.24	0.57	0.23	0.13
TRFH1	0.27	0.32	1.00	0.74	0.16	0.24	0.58	0.28
CTRSH1	0.29	0.27	0.74	1.00	0.24	0.23	0.54	0.30
SWANI1	0.83	0.24	0.16	0.24	1.00	0.40	0.31	0.40
CPRSI1	0.30	0.57	0.24	0.23	0.40	1.00	0.38	0.42
TRFI1	0.23	0.23	0.58	0.54	0.31	0.38	1.00	0.59
CTRSI1	0.37	0.13	0.28	0.30	0.40	0.42	0.59	1.00

**MZ twin 2: within person**								
	SWANH2	CPRSH2	TRFH2	CTRSH2	SWANI2	CPRSI2	TRFI2	CTRSI2
SWANH2	1.00	0.39	0.25	0.20	0.87	0.33	0.20	0.31
CPRSH2	0.39	1.00	0.27	0.20	0.31	0.55	0.24	0.14
TRFH2	0.25	0.27	1.00	0.76	0.24	0.24	0.62	0.26
CTRSH2	0.20	0.20	0.76	1.00	0.19	0.20	0.53	0.27
SWANI2	0.87	0.31	0.24	0.19	1.00	0.42	0.29	0.38
CPRSI2	0.33	0.55	0.24	0.20	0.42	1.00	0.48	0.49
TRFI2	0.20	0.24	0.62	0.53	0.29	0.48	1.00	0.54
CTRSI2	0.31	0.14	0.26	0.27	0.38	0.49	0.54	1.00

**MZ twin 1–twin 2: correlations between twin 1 and twin 2**								
	SWANH1	CPRSH1	TRFH1	CTRSH1	SWANI1	CPRSI1	TRFI1	CTRSI1
SWANH2	**0.91**	0.25	0.23	0.20	0.77	0.26	0.20	0.30
CPRSH2	0.34	**0.78**	0.18	0.15	0.25	0.54	0.18	0.14
TRFH2	0.29	0.27	**0.64**	0.49	0.18	0.25	0.52	0.27
CTRSH2	0.23	0.19	0.56	**0.60**	0.22	0.21	0.43	0.22
SWANI2	0.80	0.23	0.14	0.19	**0.85**	0.36	0.29	0.35
CPRSI2	0.25	0.46	0.19	0.17	0.29	**0.74**	0.35	0.38
TRFI2	0.21	0.23	0.41	0.34	0.22	0.41	**0.63**	0.42
CTRSI2	0.36	0.10	0.15	0.18	0.33	0.36	0.42	**0.72**

Same phenotype twin 1–twin 2 correlations shown in bold

**Table 8:** ML estimates of the DZ phenotypic correlation matrix corrected for main effects of sex

**DZ twin 1: within person**								
	SWANH1	CPRSH1	TRFH1	CTRSH1	SWANI1	CPRSI1	TRFI1	CTRSI1
SWANH1	1.00	0.47	0.38	0.20	0.81	0.36	0.43	0.34
CPRSH1	0.47	1.00	0.34	0.34	0.40	0.52	0.29	0.25
TRFH1	0.38	0.34	1.00	0.77	0.45	0.39	0.65	0.41
CTRSH1	0.20	0.34	0.77	1.00	0.26	0.34	0.59	0.43
SWANI1	0.81	0.40	0.45	0.26	1.00	0.56	0.54	0.45
CPRSI1	0.36	0.52	0.39	0.34	0.56	1.00	0.49	0.50
TRFI1	0.43	0.29	0.65	0.59	0.54	0.49	1.00	0.55
CTRSI1	0.34	0.25	0.41	0.43	0.45	0.50	0.55	1.00

**DZ twin 2: within person**								
	SWANH2	CPRSH2	TRFH2	CTRSH2	SWANI2	CPRSI2	TRFI2	CTRSI2
SWANH2	1.00	0.51	0.36	0.37	0.83	0.41	0.39	0.25
CPRSH2	0.51	1.00	0.32	0.29	0.46	0.56	0.24	0.27
TRFH2	0.36	0.32	1.00	0.75	0.36	0.32	0.64	0.37
CTRSH2	0.37	0.29	0.75	1.00	0.41	0.30	0.57	0.40
SWANI2	0.83	0.46	0.36	0.41	1.00	0.56	0.41	0.31
CPRSI2	0.41	0.56	0.32	0.30	0.56	1.00	0.47	0.46
TRFI2	0.39	0.24	0.64	0.57	0.41	0.47	1.00	0.58
CTRSI2	0.25	0.27	0.37	0.40	0.31	0.46	0.58	1.00

**DZ twin 1–twin 2 correlations between twin 1 and twin 2**								
	SWANH1	CPRSH1	TRFH1	CTRSH1	SWANI1	CPRSI1	TRFI1	CTRSI1
SWANH2	**0.46**	0.09	0.21	0.01	0.43	0.13	0.28	0.17
CPRSH2	0.13	**0.32**	0.08	0.08	0.14	0.24	0.10	0.11
TRFH2	0.11	0.08	**0.25**	0.24	0.08	0.04	0.11	0.19
CTRSH2	0.11	0.04	0.25	**0.31**	0.08	0.05	0.18	0.24
SWANI2	0.45	0.13	0.18	0.00	**0.40**	0.11	0.26	0.14
CPRSI2	0.13	0.24	0.11	0.10	0.08	**0.24**	0.14	0.13
TRFI2	0.02	0.05	0.17	0.15	0.04	0.04	**0.16**	0.24
CTRSI2	0.10	0.08	0.17	0.16	0.10	0.14	0.20	**0.41**

Same phenotype twin 1–twin 2 correlations shown in bold

## Appendix 2

See Table 9.

**Table 9:** Estimates of variance components (σ^2^_AM_, σ^2^_DM_, σ^2^_EM_) in males minus the estimates of variance components (σ^2^_AF_, σ^2^_DF_, σ^2^_EF_) in females obtained in univariate analyses

Difference	SWAN HYP	CPRS HYP	TRF HYP	CTRS HYP	SWAN INATT	CPRS INATT	TRF INATT	CTRS INATT
σ^2^_AM_–σ^2^_AF_	− 0.044 (0.388)	− 0.160 (0.167)	0.370 (0.355)	0.550 (0.323)	0.120 (0.394)	− 0.234 (0.341)	0.032 (0.080)	0.000^§^
σ^2^_DM_–σ^2^_DF_	0.122 (0.368)	0.421* (0.163)	− 0.154 (0.375)	− 0.422 (0.304)	0.065 (0.371)	0.519* (0.193)	0.000^§^	0.159 (0.089)
σ^2^_EM_–σ^2^_EF_	0.038 (0.024)	0.022 (0.047)	0.115 (0.072)	0.201 (0.112)	0.056 (0.038)	− 0.100 (0.048)	0.035 (0.061)	0.071 (0.071)

Robust standard errors in parentheses

^§^Estimated at zero (i.e., not fixed to zero)

*p < 0.01

## Appendix 3

See Table 10.

**Table 10:** Standardized variance components obtained in the multivariate Cholesky decomposition (corrected for teacher rater and sex)

	SWAN HYP	CPRS HYP	TRF HYP	CTRS HYP	SWAN INATT	CPRS INATT	TRF INATT	CTRS INATT
A	0.580	0.094	0.241	0.284	0.495	0.132	0.111	0.613
D	0.332	0.712	0.411	0.351	0.342	0.621	0.583	0.184
A + D	0.912	0.806	0.652	0.634	0.838	0.752	0.694	0.797
E	0.088	0.194	0.348	0.366	0.162	0.248	0.306	0.203

Results based on the untransformed scales

## Appendix 4

See Table 11.

**Table 11:** A + D and E correlation matrices based on the multivariate Cholesky model as fitted to the untransformed scales

A + D	SWAN H	CPRS H	TRF H	CTRS H	SWAN I	CPRS I	TRF I	CTRS I
SWAN H	***0.912***	***0.879***	***0.960***	***0.924***	***0.921***	***0.854***	***0.920***	***0.929***
CPRS H	0.526	***0.806***	***0.746***	***0.766***	***0.875***	***0.823***	***0.820***	***0.877***
TRF H	0.452	0.472	***0.652***	***0.706***	***0.816***	***0.798***	***0.747***	***0.870***
CTRS H	0.458	0.534	0.925	***0.634***	***0.916***	***0.838***	***0.712***	***0.895***
SWAN I	0.865	0.486	0.418	0.513	***0.838***	***0.805***	***0.847***	***0.849***
CPRS I	0.421	0.652	0.447	0.446	0.558	***0.752***	***0.882***	***0.843***
TRF I	0.361	0.382	0.721	0.599	0.464	0.631	***0.694***	***0.822***
CTRS I	0.327	0.248	0.381	0.320	0.394	0.531	0.636	***0.797***

E	SWAN H	CPRS H	TRF H	CTRS H	SWAN I	CPRS I	TRF I	CTRS I
SWAN H	***0.088***	***0.121***	***0.040***	***0.076***	***0.079***	***0.146***	***0.080***	***0.071***
CPRS H	0.473	***0.194***	***0.254***	***0.234***	***0.125***	***0.177***	***0.180***	***0.123***
TRF H	0.084	0.448	***0.348***	***0.294***	***0.184***	***0.202***	***0.253***	***0.130***
CTRS H	0.160	0.438	0.694	***0.366***	***0.084***	***0.162***	***0.288***	***0.105***
SWAN I	0.541	0.323	0.294	0.141	***0.162***	***0.195***	***0.153***	***0.151***
CPRS I	0.404	0.498	0.269	0.197	0.536	***0.248***	***0.118***	***0.157***
TRF I	0.153	0.257	0.504	0.481	0.286	0.221	***0.306***	***0.178***
CTRS I	0.159	0.141	0.155	0.098	0.315	0.341	0.412	***0.203***

The upper triangular part of the matrices A + D and E contain the proportion of phenotypic variance (diagonals; broad-sense heritabilities in the case of the A + D matrix; bold italics underscored), and the proportion of phenotypic covariances (above the diagonal; bold italics) explained by A + D and E

*H* hyperactivity, *I*  inattention
